# Ability of ^18^F-FDG PET/CT Radiomic Features to Distinguish Breast Carcinoma from Breast Lymphoma

**DOI:** 10.1155/2019/4507694

**Published:** 2019-02-25

**Authors:** Xuejin Ou, Jian Wang, Ruofan Zhou, Sha Zhu, Fuwen Pang, Yi Zhou, Rong Tian, Xuelei Ma

**Affiliations:** ^1^West China School of Medicine, West China Hospital, Sichuan University, No. 37 Guoxue Alley, Chengdu 610041, China; ^2^State Key Laboratory of Biotherapy and Cancer Center, West China Hospital, Sichuan University, Chengdu 610041, China; ^3^School of Computer Science, Nanjing University of Science and Technology, No. 200, Xiaolinwei Road, Nanjing 210094, China; ^4^Department of Nuclear Medicine, West China Hospital, Sichuan University, No. 37 Guoxue Alley, Chengdu 610041, China; ^5^Department of Biotherapy, West China Hospital and State Key Laboratory of Biotherapy, Sichuan University, Chengdu 610041, China

## Abstract

*Purpose*. To investigate the value of SUV metrics and radiomic features based on the ability of ^18^F-FDG PET/CT in differentiating between breast lymphoma and breast carcinoma. *Methods*. A total of 67 breast nodules from 44 patients who underwent ^18^F-FDG PET/CT pretreatment were retrospectively analyzed. Radiomic parameters and SUV metrics were extracted using the LIFEx package on PET and CT images. All texture parameters were divided into six groups: histogram (HISTO), SHAPE, gray-level co-occurrence matrix (GLCM), gray-level run-length matrix (GLRLM), neighborhood gray-level different matrix (NGLDM), and gray-level zone-length matrix (GLZLM). Receiver operating characteristics (ROC) curves were generated to evaluate the discriminative ability of each parameter, and the optimal parameter in each group was selected to generate a new predictive variable by using binary logistic regression. PET predictive variable, CT predictive variable, the combination of PET and CT predictive variables, and SUV_max_ were compared in terms of areas under the curve (AUCs), sensitivity, specificity, and accuracy. *Results*. Except for SUVmin (*p*=0.971), the averages of FDG uptake metrics of lymphoma were significantly higher than those of carcinoma (*p* ≤ 0.001), with the following median values: SUV_mean_, 4.75 versus 2.38 g/ml (*P* < 0.001); SUV_std_, 2.04 versus 0.88 g/ml (*P*=0.001); SUV_max_, 10.69 versus 4.76 g/ml (*P*=0.001); SUV_peak_, 9.15 versus 2.78 g/ml (*P* < 0.001); TLG, 42.24 versus 9.90 (*P* < 0.001). In the ROC curves analysis based on radiomic features and SUV_max_, the AUC for SUV_max_ was 0.747, for CT texture parameters was 0.729, for PET texture parameters was 0.751, and for the combination of CT and PET texture parameters was 0.771. *Conclusion*. The SUV metrics in ^18^FDG PET/CT images showed a potential ability in the differentiation between breast lymphoma and carcinoma. The combination of SUV_max_ and PET/CT texture analysis may be promising to provide an effectively discriminant modality for the differential diagnosis of breast lymphoma and carcinoma, even for the differentiation of subtypes of lymphoma.

## 1. Introduction

Breast nodules have been investigated with many imaging technologies, including ultrasonography, mammography, and MRI, PET/CT. Great progress has been made in functional imaging technology in the past decade, allowing the screening for tumors to be noninvasive and efficient. With the development of these new functional technologies, it is becoming easier and easier for radiologists to distinguish malignant lesions from benign ones. Furthermore, the diagnostic sensitivity and specificity of imaging technologies for tumors have been improved dramatically at the same time. However, as discussed above, current studies on the use of imaging technologies mainly focus on the differentiation between benign and malignant lesions [1–3]. Mostly, malignant nodules in the breast were considered as breast carcinoma at the time of diagnosis, while breast lymphoma was usually neglected due to its rarity.

Primary breast lymphoma makes up about 2.2% of extranodal lymphomas and accounts for less than 0.5% of breast malignancies. Although breast lymphoma is relatively uncommon, the incidence of breast lymphoma has been increasing over the last four decades and will continue to increase in younger women [4]. The clinical and imaging presentations of breast lymphoma mimic those of breast carcinoma. They both present as painless breast mass, occasionally accompanied with erythema and edema in physical examination, and both show solitary mass with irregular margin on images [5, 6]. Fine needle biopsy is considered the golden standard for the diagnosis of breast cancer, while the pathological diagnosis of breast lymphoma is challenging because of its rare incidence and its difficulty in differentiating the lymphoid cells from reactive lymphocytes in the core needle biopsy samples [7]. Due to the diagnostic difficulties and great differences lying in the treatments of breast carcinoma and breast lymphoma, mistakes could be made frequently in the management of these two different breast malignancies. Surgery is the main treatment in the management of early stage breast carcinoma [8], but for patients with breast lymphoma, mastectomy has been reported to offer no benefits, or even bring poor survival [9, 10]. However, in this retrospective research, we found that about 55% patients with breast lymphoma were misdiagnosed as breast carcinoma and received inappropriate mastectomy. Furthermore, the clinical outcome of these two breast malignancies differs a lot. Since the introduction of rituximab, the prognosis of lymphoma has been improved significantly [11–14].

Therefore, it is critical for clinical doctors to distinguish breast lymphoma from breast cancer since their management differs a lot. Texture analysis has been reported to be an efficient tool to quantify tissue gray-level patterns, pixel interrelationships, spectral properties, and tumor heterogeneity [15, 16]. Studies have reported that texture analysis applied in computed tomography (CT), ultrasonography (US), and magnetic resonance imaging (MRI) could provide better differentiation between benign and malignant lesions in the breast [17, 18], lung [19], and kidney [20, 21]. Recently, texture analysis of ^18^F-FDG PET/CT have been conducted on differentiating nodules, and it showed better diagnostic value than previous routine parameters such as SUV metrics [22]. Also, a research showed that PET radiomic features could be promising for identification of primary and metastatic lung cancers [23]. While another research reported that the SUV metrics extracted from PET/CT images might be useful in differentiating renal cell carcinomas from renal lymphomatous involvement [24, 25]. Hence, we hypothesized that the combination of texture analysis and SUV metrics on ^18^F-FDG PET/CT in differentiating cancer and lymphoma in the breast might be useful in the future. In the present research, we sought to investigate the value of SUV metrics and texture analysis based on ^18^F-FDG PET/CT in differentiating breast carcinoma and breast lymphoma.

## 2. Methods

### 2.1. Study Population

In this retrospective study, we analyzed 67 breast nodules from 44 patients who underwent ^18^F-FDG PET/CT for staging between October 2013 and March 2018 at West China Hospital, Sichuan University. Patients with pathological diagnosis of breast lymphoma or breast carcinoma were included. Patients were excluded when having (1) any kinds of treatment before ^18^F-FDG PET/CT scanning including surgery, chemotherapy, and radiotherapy; (2) inconclusive diagnosis due to inadequate biopsy sample; and (3) other types of cancers apart from breast cancer and lymphoma. A total of 44 patients (19 patients with breast lymphoma and 25 patients with breast carcinoma) were included in our present study. For all patients, available patients' clinical characteristics are summarized in Table 1. This study was approved by Medical Ethics Committee, Sichuan University. All procedures were conducted in accordance with the Declaration of Helsinki and its later amendments. Written informed consent was provided by every participant.

### 2.2. Image Acquisition and Texture Analysis

Whole-body 18F-FDG PET/CT examinations were administrated in all patients on a Gemini GXL PET/CT scanner equipped with a 16-slice CT (Philips Medical System, Cleveland, Ohio, USA). All patients fasted for at least 6 h before intravenous injection of 190–375 MBq of ^18^F-FDG (5.18 MBq/kg). Blood glucose levels of all patients were controlled to be lower than 8.0 mmol/L at the time of examination. A low-dose CT (5 mm slice thickness; tube voltage, 120 kV; tube current, 40 mAs) was performed for attenuation correction, immediately followed by PET emission scan without changing the position of patients. PET and CT images were acquired from head to extremities. Radiomic parameters and SUV metrics extraction of the breast nodules were performed using the LIFEx package [26] on PET and CT images. Whole-body PET/CT images were interpreted and delineated by an experienced radiologist who was blind to patients' clinical and pathological information. The volume-of-interest (VOI) was manually contoured in all subsequent slices on PET images, and the same VOI was applied on CT images. Before extracting parameters, intensity discretization was automatically performed by the software with the number of gray levels of 64 bins and the intensity rescaling was defined as absolute scale bounds between 0 and 20 for PET images. Similarly, intensity discretization for CT images was performed with the number of gray levels of 400 bins and absolute scale bounds between −1000 and 3000 HU. Features including conventional SUV metrics (SUV_min_, SUV_mean_, SUV_max_, SUV_peak_, and TLG) and radiomic parameters (both first and second order features) were extracted from PET and CT images, respectively, with the whole layers in 3D volume-of-interest (VOI). The mathematical definitions of features are summarized in Supplementary Table 1.

### 2.3. Statistical Analysis

Since not all of the radiomic parameters were helpful for the differential diagnosis of breast lymphoma and breast carcinoma, we divided these parameters into six groups: histogram (HISTO), SHAPE, gray-level co-occurrence matrix (GLCM), gray-level run-length matrix (GLRLM), neighborhood gray-level different matrix (NGLDM), and gray-level zone-length matrix (GLZLM) group. According to the performances of these parameters in the receiver operating characteristics (ROC) curves test, the most discriminative parameters in each group were selected, as shown in Table 2. Since the gross heterogeneity of a tumor is consisted of multiple texture patterns, a single parameter is not sufficient to describe its whole characteristics. A new predictive variable (a combination of the six optimal parameters on PET or CT images) was obtained by using binary logistic regression. Thus, besides conventional SUV metrics, we developed three more discriminative methods: two of which were based on the optimal parameters extracted from PET or CT images, and the third is the combination of both PET and CT texture parameters (Table 3).

The differences between breast lymphoma and carcinoma in each SUV metric were evaluated using the Mann–Whitney *U* test. A comparing ROC analysis was conducted on SUVmax, CT predictive variable, PET predictive variable, and combined CT and PET predictive variables. The area under the curve (AUC) was used to assess the discriminative ability of each method. All statistical analyses were performed using SPSS Statistics Version 22.0 and MedCalc (MedCalc Software bvba, Acacialaan, Belgium). *P* value < 0.05 was considered significant.

## 3. Results

### 3.1. Patient Characteristics

Forty-four patients with sixty-seven nodules were included in our study. All of the patients were women with a median age of 55.5 years old (range from 26 to 80 years old). Of the 44 patients, 25 (56.82%) were with breast carcinoma, and the subtypes of carcinoma included 18 invasive ductal carcinomas, 2 invasive lobular breast carcinomas, 3 ductal carcinomas in situ (DCIS) of breast, and 2 other special subtypes of breast carcinoma. The remaining 19 (43.18%) nodules were identified as lymphomas, 15 of the breast lymphomas were diffuse large B cell lymphoma, 2 were NK/T cell lymphoma, 1 was a Hodgkin lymphoma, 1 was a intravascular B cell lymphoma (Table 1).

### 3.2. FDG Uptake Metrics

The results of ROC analysis of FDG uptake metrics for breast lymphoma versus breast carcinoma are summarized in Table 4. Except for SUV_min_ (*p*=0.971), all the averages of FDG uptake metrics of lymphoma were significantly higher than that of carcinoma (*p* ≤ 0.001), with the following median values: SUV_mean_, 4.75 versus 2.38 g/ml (*P* < 0.001); SUV_std_, 2.04 versus 0.88 g/ml(*P*=0.001); SUV_max_, 10.69 versus 4.76 g/ml (*P*=0.001); SUV_peak_, 9.15 versus 2.78 g/ml (*P* < 0.001); TLG, 42.24 versus 9.90 (*P* < 0.001). Significant diagnostic value could be found in SUV_mean_ with an AUC of 0.747 (*P*=0.001); in SUV_std_ with an AUC of 0.755 (*P* < 0.001); in SUV_max_ with an AUC of 0.747 (*P*=0.001); in SUV_peak_ with an AUC of 0.749 (*P* < 0.001); in TLG with an AUC of 0.754 (*P* < 0.001), whereas no significance was observed in SUV_min_ (AUC, 0.437, *p*=0.379).

### 3.3. Radiomic Parameters

The three predictive models constructed using optimal parameters from each group by binary logistic regression were as follows:

#### 3.3.1. PET Predictive Model

(1) $$\begin{array}{c} {\text{PRE}}_{\text{PET}}=-2.380\text{HISTO}\_\text{Entropy}\_\text{log}10-0.001\text{SHAPE}\_\text{Volume}\left(\text{VX}\right)+0.093\text{GLCM}\_\text{Entropy}\_\text{log}10-0.013\text{GLRLM}\_\text{HGRE}-2.085\text{NGLDM}\_\text{Contrast}+0.016\text{GLZLM}\_\text{HGZE}+2.903. \end{array}$$

#### 3.3.2. CT Predictive Model

(2) $$\begin{array}{c} {\text{PRE}}_{\text{CT}}=-0.012\text{HISTO\_Kurtosis}-0.057\text{SHAPE\_Volume}\left(\text{mL}\right)+1.277\text{GLCM\_Homogeneity}+0.001\text{GLRLM\_RLNU}-0.472\text{NGLDM\_Busyness}+5.31\times 10^{-9}\text{GLZLM\_LZHGE}+0.246. \end{array}$$

#### 3.3.3. Combined Predictive Model

(3) $$\begin{array}{c} {\text{PRE}}_{\text{combination}}=0.256\text{SUVmax}-2.505\text{HISTO}\_\text{Entropy}\_\text{log}10-0.001\text{SHAPE}\_\text{Volume}\left(\text{VX}\right)-0.294\text{GLCM}\_\text{Entropy}\_\text{log}10-0.015\text{GLRLM}\_\text{HGRE}-3.674\text{NGLDM}\_\text{Contrast}+0.015\text{GLZLM}\_\text{HGZE}+2.468-0.006\text{HISTO}\_\text{Kurtosis}-0.011\text{SHAPE}\_\text{Volume}\left(\text{mL}\right)+1.772\text{GLCM}\_\text{Homogeneity}+2.15\times 10^{-4}\text{GLRLM\_RLNU}-0.457\text{NGLDM}\_\text{Busyness}+1.36\times 10^{-9}\text{GLZLM}\_\text{LZHGE.} \end{array}$$

**Figure 1:**
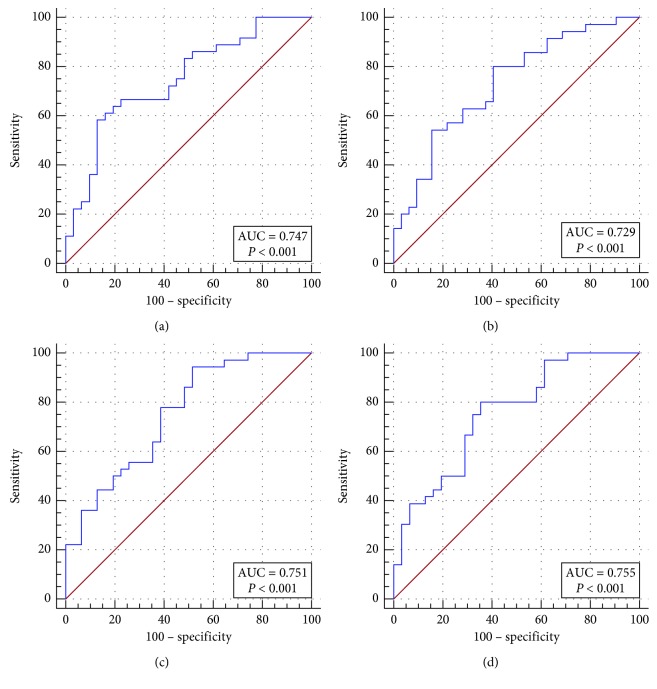
ROC curves of the three radiomic predictive models and SUV method in differentiating breast lymphoma and breast carcinoma.

Table 5 displays a comparison of differential diagnostic ability among the three radiomic predictive variables and the SUV method (see also in Figure 1). The SUV method showed a sensitivity of 61.11%, specificity of 87.10%, and accuracy of 70.15%; compared with the SUV method, CT and PET predictive variables both showed an improved sensitivity (80.00% and 94.44%), but a decreased specificity (59.38% and 48.39%). The combination of CT and PET predictive variables showed a relatively better performance, with sensitivity of 83.33%, specificity of 64.52%, and accuracy of 74.63%, compared with the SUV method (*p* < 0.0001). Among the four diagnostic models, the PET predictive variable showed the highest sensitivity but the lowest specificity. On the contrary, the SUVmax method showed the highest specificity but the lowest sensitivity.

**Figure 2:**
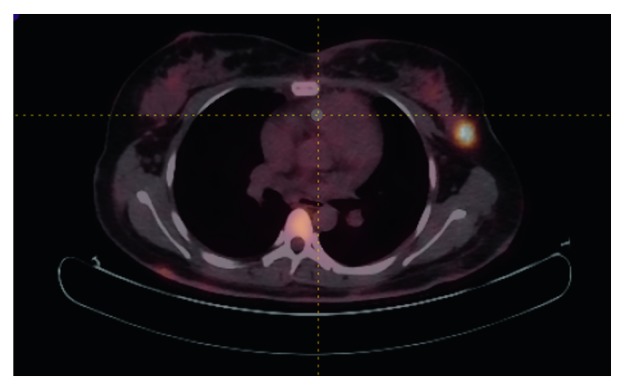
An example of lymphoma that was misdiagnosed as carcinoma by the three radiomic predictive models and the SUV method (SUV_max_ = 6.4, PET radiomic parameters: HISTO_Entropy_log10 = 1.14, SHAPE_volume (# vx) = 183, GLCM_Entropy_log10 = 1.95, GLRLM_HGRE = 99.7, NGLDM_contrast = 0.147, GLZLM_HGZE = 143.1; CT radiomic parameters: HISTO_kurtosis = 1.92 SHAPE_volume (mL) = 11.380, GLCM_homogeneity = 0.453, GLRLM_RLNU = 1119.4, NGLDM_busyness = 0.903, GLZLM_LZHGE = 4040958.550).

**Figure 3:**
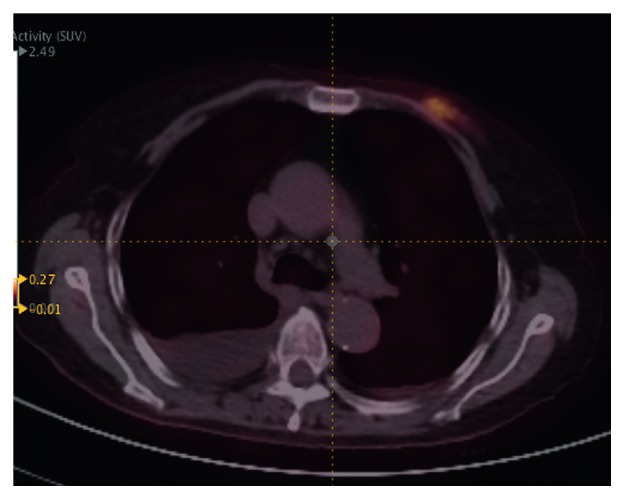
An example of lymphoma that was misdiagnosed as carcinoma by the three radiomic predictive models and the SUV method (SUV_max_ = 2.1, PET radiomic parameters: HISTO_Entropy_log10 = 0.74, SHAPE_volume (# vx) = 34, GLCM_Entropy_log10 = 1.07, GLRLM_HGRE = 17.1, NGLDM_contrast = 0.158, GLZLM_HGZE = 17.5; CT radiomic parameters: HISTO_kurtosis = 21.11. SHAPE_volume (mL) = 2.325, GLCM_homogeneity = 0.382, GLRLM_RLNU = 279.2, NGLDM_busyness = 0.050, GLZLM_LZHGE = 183718.048).

**Figure 4:**
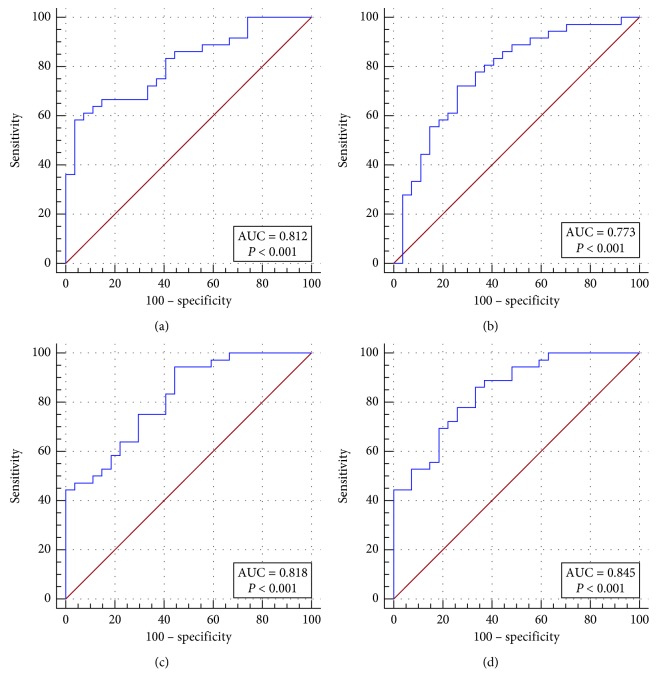
Comparison of the discriminant ability among the three radiomic predictive models and the SUV method in breast DLBCL and carcinoma.

Figures 2 and 3 are two examples of lymphoma that were misdiagnosed as carcinoma by all the three radiomic predictive models and the SUV method. The FDG uptakes in these two cases were relatively low, and the values of PET and CT radiomic parameters were similarly to that of carcinoma. Besides, we found another two cases which were also misclassified by all of the four differential diagnosis methods. And then, we checked their pathological subtypes and found that their pathological identifications were NK/T cell lymphoma, Hodgkin's lymphoma, and intravascular B cell lymphoma. Apart from these four cases, the nodules remaining were all diffuse large B cell lymphomas. Thus, we continually compared the discriminant ability of the three radiomic models and SUV model in differentiating diffuse large B cell lymphoma and carcinoma in the breast. The Results are summarized in Table 6 and Figure 4. The AUC, sensitivity, specificity, and accuracy of all kinds of methods improved significantly, especially for the specificity.

## 4. Discussion

Our results indicated that PET/CT SUV metrics and radiomic parameters could assist in the differentiation of breast carcinoma and breast lymphoma, especially between breast carcinoma and breast diffuse large B cell lymphoma. Most SUV metrics of breast lymphoma were significantly higher than that of breast carcinoma. In the four discriminative models, SUV_max_ showed the highest specificity, the PET radiomic parameters showed the highest sensitivity, and the combination of CT and PET radiomic parameters had the best accuracy. The AUC, accuracy, and specificity of all these four models improved significantly, when they were applied in differentiating between breast DLBCL and breast carcinoma.

To our knowledge, this is the first study on the use of ^18^FDG PET/CT SUV metrics and texture analysis in the differential diagnosis of breast carcinoma and lymphoma. Daoud et al. [27] investigated 64 benign tumors and 46 malignant tumors on breast ultrasound images by using multiple-region-of-interest (ROI) texture analysis and demonstrated that the proposed texture analysis was effective in differentiating between benign and malignant tumors. Hodgdon et al. [20] found that texture analysis could accurately distinguish fat-poor angiomyolipoma from renal cell carcinoma, but failed to differentiate subtypes of renal cell carcinoma on unenhanced CT images. Using the combination of CT texture and shape features, Bayanati et al. [28] found 71% benign and 84% malignant mediastinal lymph nodes could be correctly classified. Studies on MRI texture analysis also showed radiomics could precisely differentiate among glioma subtypes [29–31]. However, most studies focus on the differentiation between benign and malignant tumors, possibly because the heterogeneity between benign and malignant tumors differs a lot. Researches in the setting of differentiation among malignant tumors and subtypes of cancers are limited. Ma et al. [32] demonstrated that CT-based radiomic signature has the potential to distinguish Borrmann type IV gastric cancer from primary gastric lymphoma. Kirienko et al. [23] proved PET radiomic features were able to classify the primary and metastatic lung lesions and even were promising for identifying subtypes of primary lung cancers. Nevertheless, the ability of radiomics based on ^18^FDG PET/CT in differentiating breast malignant lesions was not clear. Our study showed that radiomic features and conventional SUV metrics could provide a promising noninvasive tool for differential diagnosis of breast malignancies in clinical practice.

Recent studies have investigated the ability of SUV metrics (SUV_mean_ and SUV_max_) in differentiating renal cell carcinomas (RCCs) and kidney involved lymphoma and suggested that RCCs were significantly less FDG avid than renal lymphomatous involvement [24, 25]. In our study, except for SUV_min_, SUV metrics of breast lymphoma including SUV_max_, SUV_mean_, SUV_std_, and TLG were significantly higher than those of breast carcinoma, which was consistent with previous studies. In addition, SUV_max_ exhibited a much higher specificity than any of other methods in distinguishing lymphoma from carcinoma in our study. Especially in the comparison of breast DLBCL and carcinoma, the specificity was up to 96.3%.

It is still a confounding problem why different tumors show different FDG uptakes. Some researchers believe that the FDG uptake differences may be a reflection of tumor's proliferation [33, 34]. Shou et al. [35] investigated the correlation between FDG uptake and tumor-proliferating antigen Ki-67 in lymphomas. Their results suggested that greater proliferative ability was the leading cause for higher FDG uptakes, and tumors of high proliferation such as DLBCL often showed a significantly high FDG uptake; in contrast, FDG uptake was relatively low in tumors of indolent proliferation. Therefore, the differences in proliferation may be a possible explanation to the different FDG uptakes between breast carcinoma and lymphoma.

At present, the interpretation of medical images is based on qualitative criteria and is heavily dependent on the experience of radiologists. However, increasingly accumulating evidences showed that medical images contained some important information that was usually neglected by naked eyes [36]. Thus, a more subjective and quantitative way for radiographic image evaluation was needed. Radiomics as an emerging field provided such a way of objectively measuring tumor heterogeneity by quantifying underlying tissue gray-level patterns. In our study, PET radiomic features proved to be useful in distinguishing breast carcinoma from lymphoma, with a sensitivity of 94.44% and an accuracy of 71.64% (*p* < 0.0001). The previous study postulated that the texture features in PET images might reflect tumor heterogeneity associated with cell proliferation, hypoxia, necrosis, perfusion, and calcification [37]. It is well known that the biological behaviors of breast carcinoma and lymphoma differ dramatically [5, 38]. In our past investigation, lymphomas showed greater proliferative abilities but with less hemorrhage, less tissue calcification, and cell necrosis when compared with breast carcinoma.

In our findings, CT radiomic features did not perform as good as PET radiomic features, with a sensitivity of only 80.00%, specificity of 59.38%, and accuracy of 68.66% (*p* value < 0.0001). But the combination of PET and CT texture parameter could improve diagnostic accuracy up to 74.63%. Previous data indicated that the performance of CT texture analysis was better than that of PET texture analysis, but our results were in agreement with the findings of Kirienko et al. [23]; both of our results showed that the radiomic features from CT images were inferior to features from PET images in the differential diagnosis. Currently, no definitive correlation of a particular radiomic parameter with underlying biological process has been investigated clearly. According to the results in our research, we speculated that PET texture features could still provide additional information to CT texture analysis on tumor's metabolism even after SUV metrics were excluded from the PET texture data.

^18^FDG PET/CT is a promising modality for functional imaging in evaluating tumor metabolism. SUV_max_ is the most commonly used parameter in metabolism evaluation in the clinical routine. In our results, SUV_max_ exhibited the highest specificity in differentiating between lymphoma and breast carcinoma. The PET texture parameters possessed the highest sensitivity, while the combination of CT and PET texture features showed the best accuracy. Thus, we speculated that the combination of SUV_max_ and texture analysis of PET/CT images might be a potentially promising and noninvasive tool in the differential diagnosis of breast carcinoma and lymphoma. SUV_max_ could provide information on tumor metabolism, while the PET and CT radiomic features could show spatial variation of tumor structure and tumor heterogeneity, which may not be perceived by naked eyes. Moreover, the texture analysis is a computational postprocessing technique, and data could be acquired during routinely clinical imaging protocols; additionally, useful information could be extracted without increasing cost for the healthcare system. Furthermore, although biopsy is considered as the golden standard for the diagnosis of breast nodules, small samples of biopsy are not always reliable in the diagnosis of lymphoma. In addition, the differentiation of newly found breast nodules in patients with previous lymphoma or cancer history is really challenging. Therefore, the conventional FDG uptake parameter SUV_max_ combined with PET/CT texture analysis may help a lot in this situation.

However, there are several limitations in our study we have to admit. Firstly, this is a retrospective and single-institutional study. Secondly, as breast lymphoma is a rare entity, it could not be frequently encountered. Moreover, we only included patients who underwent ^18^FDG PET/CT scanning before receiving any treatments, so we had a relatively small sample. Thirdly, as DLBCL is the predominant histological subtype of breast lymphoma, accounting for 78.95% in our study, only 4 cases of other subtypes were included apart from DLBCL. This, to some extent, may lead to bias in the comparison of breast DLBCL and carcinoma. But, the potential value of SUV_max_ and PET texture parameters were still obvious in differentiating DLBCL from breast carcinoma, or even in the characterization of subtypes of lymphoma.

In conclusion, the SUV metrics in ^18^FDG PET/CT images showed a potential ability in the differentiation of breast lymphoma and carcinoma. And, the combination of SUV_max_ and PET/CT texture analysis may be promising to provide an effectively discriminant modality for the differential diagnosis of breast lymphoma and carcinoma and even for the differentiation of subtypes of lymphoma. Further investigation and validation with a larger sample is warranted to confirm our findings.

## Figures and Tables

**Table 1:** Patient characteristics.

Characteristic	Value
Age (y), median (range)	55.5 (26–80)

Sex	
Male	0
Female	44

Histologic findings	
Hodgkin's lymphoma	1
Diffuse large B-cell lymphoma	15
Intravascular large B-cell lymphoma	1
NK/T-cell lymphoma	2
Invasive ductal carcinoma	18
Invasive lobular breast carcinoma	2
Ductal carcinoma in situ (DCIS) of breast	3
Other special subtypes of breast carcinoma	2

**Table 2:** The results of ROC analysis of potentially optimal texture parameters in PET and CT images for breast lymphoma versus breast carcinoma.

Variable	Lymphoma		Carcinoma		AUC	*P* value
	Median	Range	Median	Range		
Radiomic parameters on PET						
HISTO_entropy_log10	1.36	0.64–1.76	0.97	0.32–1.66	0.740	0.001
SHAPE_volume(# vx)	183.00	22.00–6334.00	71.00	11.00–4241.00	0.725	0.002
GLCM_entropy_log10	2.04	1.00–3.19	1.54	0.59–3.03	0.730	0.001
GLRLM_HGRE	287.95	17.14–1919.15	75.66	9.96–862.28	0.752	<0.001
NGLDM_Contrast	0.33	0.04–2.32	0.16	0.00–0.59	0.712	0.003
GLZLM_HGZE	266.62	17.53–1593.64	83.77	9.67–832.22	0.754	<0.001

Radiomic parameters on CT						
HISTO_Kurtosis	5.02	1.90–217.17	2.84	1.49–97.34	0.660	0.024
SHAPE_volume (mL)	11.38	1.07–403.07	4.40	0.73–272.41	0.728	0.001
GLCM_Homogeneity	0.49	0.08–0.60	0.44	0.33–0.65	0.628	0.072
GLRLM_RLNU	977.06	135.49–24346.21	507.35	80.31–19527.32	0.685	0.009
NGLDM_Busyness	0.53	0.04–6.04	0.38	0.13–2.77	0.602	0.152
GLZLM_LZHGE	14709543.63	77721.12–1603305720.99	1385779.22	44958.07–435410392.46	0.681	0.011

Abbreviations: HISTO, histogram; GLCM, gray-level co-occurrence matrix; GLRLM, gray-level run-length matrix; HGRE, high gray-level run emphasis; GLZLM, gray-level zone-length matrix; HGZE, high gray-level zone emphasis; RLNU, run-length nonuniformity; LZHGE, long-zone high gray-level emphasis.

**Table 3:** Parameters used in each differential model.

Method	Parameters	
SUV metrics	SUV_max_	

CT predicted variable	HISTO_kurtosis	SHAPE_volume (mL)
	GLCM_homogeneity	GLRLM_RLNU
	NGLDM_busyness	GLZLM_LZHGE

PET predicted variable	HISTO_entropy_log10	SHAPE_volume(# vx)
	GLCM_entropy_log10	NGLDM_contrast
	GLRLM_HGRE	GLZLM_HGZE

Combination of PET and CT texture parameters	HISTO_kurtosis	SHAPE_volume (mL)
	GLCM_homogeneity	GLRLM_RLNU
	NGLDM_busyness	GLZLM_LZHGE
	HISTO_entropy_log10	SHAPE_volume(# vx)
	GLCM_entropy_log10	NGLDM_contrast
	GLRLM_HGRE	GLZLM_HGZE

Abbreviations: HISTO, histogram; GLCM, gray-level co-occurrence matrix; GLRLM, gray-level run-length matrix; HGRE, high gray-level run emphasis; GLZLM, gray-level zone-length matrix; HGZE, high gray-level zone emphasis; RLNU, run-length nonuniformity; LZHGE, long-zone high gray-level emphasis.

**Table 4:** The results of ROC analysis of FDG uptake metrics for breast lymphoma versus breast carcinoma.

Variable	Lymphoma		Carcinoma		*P* ^a^ value	AUC	*P* ^b^ value
	Median	Range	Median	Range			
SUV_min_	0.80	0.12–2.84	0.96	0.35–1.77	0.971	0.437	0.379
SUV_mean_	4.75	1.03–17.02	2.38	0.79–8.29	<0.001	0.747	0.001
SUV_std_	2.04	0.33–12.30	0.88	0.15–3.61	0.001	0.755	<0.001
SUV_max_	10.69	2.10–43.21	4.76	1.05–18.43	0.001	0.747	0.001
SUV_peak_	9.15	0.00–40.65	2.78	0.00–16.33	<0.001	0.749	<0.001
TLG	42.24	2.24–4031.99	9.90	0.55–2240.40	0.001	0.754	<0.001

*Note. P* ^a^ refers to the significance for ROC curves, *P* ^b^ is for Mann–Whitney *U* tests between breast lymphoma and breast carcinoma.

**Table 5:** Comparison of differential diagnostic ability of the three predictive models and the SUV methods.

	AUC	Sensitivity (%)	Specificity (%)	Accuracy (%)	*P* value
SUV_max_	0.747	61.11	87.10	70.15	<0.0001
CT predictive variable	0.729	80.00	59.38	68.66	0.0002
PT predictive variable	0.751	94.44	48.39	71.64	<0.0001
CT combined with PT predictive variables	0.771	83.33	64.52	74.63	<0.0001

**Table 6:** Comparison of discriminant ability of the three radiomic predictive and the SUV method in breast DLBCL and carcinoma.

	AUC	Sensitivity (%)	Specificity (%)	Accuracy (%)	*P* value
SUV_max_	0.812	58.30	96.3	73.02%	<0.0001
CT predictive variable	0.773	72.22	74.07	74.19%	<0.0001
PT predictive variable	0.818	94.44	55.56	76.19%	<0.0001
CT combined with PT predictive variables	0.845	86.11	66.67	76.19%	<0.0001

## Data Availability

The data used to support the findings of this study are available from the corresponding author upon request.

## Supplementary Materials

Supplementary Materials Supplementary Table 1: radiomic features extracted from PET and CT images and mathematical definitions for features assessed in the study.
